# EGF, TGF-*α* and Amphiregulin Differently Regulate Endometrium-Derived Mesenchymal Stromal/Stem Cells

**DOI:** 10.3390/ijms241713408

**Published:** 2023-08-29

**Authors:** Rimma Sergeevna Kamentseva, Marianna Viktorovna Kharchenko, Gulnara Vladikovna Gabdrahmanova, Michael Alexandrovich Kotov, Vera Vladislavovna Kosheverova, Elena Sergeevna Kornilova

**Affiliations:** 1 Institute of Cytology of the Russian Academy of Sciences, Tikhoretsky Ave. 4, St. Petersburg 194064, Russia; mariannakharchenko@gmail.com (M.V.K.); vera77867@mail.ru (V.V.K.); elena.kornilova@gmail.com (E.S.K.); 2 Institute of Biomedical Systems and Biotechnology, Peter the Great St. Petersburg Polytechnic University, Hlopina St. 11, St. Petersburg 195251, Russia; 3 Faculty of Biology, St. Petersburg State University, 7-9 Universitetskaya Embankment, St. Petersburg 199034, Russia

**Keywords:** epidermal growth factor receptor, EGF, transforming growth factor-α, amphiregulin, mesenchymal stromal/stem cells, HeLa, proliferation, differentiation, endometrium

## Abstract

The prototypical receptor tyrosine kinase epidermal growth factor receptor (EGFR) is regulated by a set of its ligands, which determines the specificity of signaling and intracellular fate of the receptor. The EGFR signaling system is well characterized in immortalized cell lines such as HeLa derived from tumor tissues, but much less is known about EGFR function in untransformed multipotent stromal/stem cells (MSCs). We compared the effect of epidermal growth factor (EGF), transforming growth factor-α (TGF-α) and amphiregulin (AREG) on physiological responses in endometrial MSCs (enMSC) and HeLa cells. In addition, using Western blotting and confocal microscopy, we studied the internalization and degradation of EGFR stimulated by the three ligands in these cell lines. We demonstrated that unlike HeLa, EGF and TGF-α, but not AREG, stimulated enMSC proliferation and prevented decidual differentiation in an EGFR-dependent manner. In HeLa cells, EGF targeted EGFR for degradation, while TGF-α stimulated its recycling. Surprisingly, in enMSC, both ligands caused EGFR degradation. In both cell lines, AREG-EGFR internalization was not registered. In HeLa cells, EGFR was degraded within 2 h, restoring its level in 24 h, while in enMSC, degradation took more than 4–8 h, and the low EGFR level persisted for several days. This indicates that EGFR homeostasis in MSCs may differ significantly from that in immortalized cell lines.

## 1. Introduction

Multipotent stromal/stem cells (MSCs) are of great interest for various therapeutic applications, including regenerative and reparative medicine, as well as anticancer therapy. Intensive studies in recent years have been focused on the analysis of various aspects of MSCs functioning. As a result, the idea of the key role of Wnt-, Notch-, TGF-β and FGF-signaling pathways as specific for MSCs has been formulated [1,2,3]. However, attention has also been paid to such canonical signaling system as the epidermal growth factor receptor (EGFR) family and their ligands. The involvement of the EGF receptor in embryonic development and tissue maturation has also been reported using embryonic cell lines and EGFR-knockout mice models [4,5,6,7,8], which indicates its essential role in the vital activity of pluripotent stem cells. Expression of this prototypical tyrosine kinase receptor and some of its ligands was also reported in MSCs of various tissue origin, including endometrium [9,10,11,12]. Studies on the EGFR role in endometrial MSC (enMSC) provide several lines of evidence of its participation in the regulation of both proliferation and differentiation into decidual cells that are essential for embryo implantation. However, while this fact is established, the molecular mechanisms of its implementation in enMSCs, especially in the aspect of coordination with other signals, are not fully understood. Endometrium is a tissue consisting of complexly organized layers of several types of cells undergoing transformations during the menstrual cycle under the control of estrogen and progesterone that orchestrate several signaling networks, including the EGFR-dependent one [13,14]. However, the final picture is not quite clear, and the results obtained are rather controversial.

This uncertainty may be due in part to the models and protocols used for analysis of EGFR, as well as its ligand expression and function, for example, biopsy samples or cultivated enMSC lines, histological immunostaining or mRNA assays. All researchers agree that EGFR system components are expressed in the endomerium, but in the case of biopsies, EGFR expression is often found in the epithelial layers of endometrium and not in stromal cells [9], though there are also data on the stromal expression of EGFR [15]. Different spatial expression patterns were reported for other members of the EGFR family. The data on the spatiotemporal expression of several EGFR ligands diverge significantly as well [9,14,15,16]. EGFR and some of its ligands were also detected in isolated stromal enMSC lines [17]. The temporal pattern and the way of EGFR action are ambiguous: maximal EGFR expression was shown in the secretory phase of the cycle and the key role of EGFR in decidualization and implantation processes was demonstrated in human and mice models [6,17], while other researchers reported expression of EGFR in proliferating endometrium, as well as its rise in proliferative phase in correlation with estrogen but not progesterone level [14,18,19]. It is also known that the essential components of serum-free media of defined composition used for prolonged cultivation of proliferative MSC include fibronectin, FGF2 and epidermal growth facor (EGF). In addition, the inhibitor of TGF-β, but not EGFR, was proposed to be introduced to prevent spontaneous differentiation of enMSC. This notion indicates the key role for EGFR as pro-proliferative factor [11].

In addition to the importance of studying the mechanisms of endometrium functioning for the development of infertility treatment approaches, it is also essential to be able to model the side effects on normal tissues in response to the treatment of EGFR-dependent tumors, for example, lung cancers, for which the drugs based on EGF receptor tyrosine kinase inhibitors are used [20].

Note that EGFR functions were studied for a long time on immortalized cells, obtained mostly from tissues of tumor origin. The vast majority of these lines express, according to various estimates, from hundreds of thousands (HeLa, A549) up to 1–3 million (A431) EGFR molecules per cell [21,22], which facilitates the detection of this protein both by biochemical techniques and fluorescence microscopy. However, there is strong evidence that the high level of the receptor expression is associated with malignancy and poor prognosis for a patient [23,24,25,26,27]. Usually, such cell lines depend on exogenous stimuli to a lesser extent than normal cells for several reasons. First, in such lines, even in the absence of mutations in the EGFR gene itself, the genes related to proliferation, transcription and DNA repair are often upregulated compared with normal tissues, as it was shown for cervical carcinoma line HeLa [28]. Second, high EGFR density at the cell surface as a result of the EGFR amplification/overexpression is known to stimulate receptor homo- and heterodimerization. Although ligand binding is usually expected to be required for EGFR-dependent signaling [29,30], some studies suppose that EGFR overexpression might lead to ligand-independent kinase activation [29,31,32]. Third, many tumor cells secrete such ligands as transforming growth factor-α (TGF-α), thus maintaining cell growth through the autocrine loop [33]. And finally, high amounts of EGFR protein as well as some mutations in its extra- and intracellular domains affect intracellular behavior of the receptor [34,35], changing the degradation/recycling balance in favor of the receptor recycling and consequently constantly signaling. Paradoxically, the main ideas about EGF-dependent signaling cascades organization and EGF-stimulated processes were formed on the basis of data obtained on such lines, and the molecular aspects of signaling cascades in HeLa cells are studied much better than those in MSC in general, and in enMSC in particular. On the contrary, although enMSCs can also undergo transformation, they are able to remain in the proliferative state under the control of hormones and growth factors for a long time, as well as undergo differentiation or senescence, and thus be a model of ”normal” cells while maintaining their tissue specificity.

It is known that the EGF receptor system, in addition to the EGFR itself, includes three of its orthologs belonging to the c-ErbB family of transmembrane receptor tyrosine kinases (TK), as well as a dozen of ligands with different specificities for each member of the family. Thus, for the EGF receptor (c-ErbB1), the specific ligands are EGF, TGF-α and amphiregulin (AREG). All three ligands are synthesized as membrane-bound precursors, the extracellular part of which can be converted by metalloproteases into a free soluble form [36]. Thus, these ligands can participate in all types of intercellular regulation, i.e., endocrine, paracrine, juxtacrine and autocrine. The latter is especially characteristic of TGF-α in transformed cells. AREG stands apart from the above-mentioned ligands, since its precursor in addition has a small intracellular domain capable of acting as an epigenetic transcription regulator [37], and AREG synthesis is estrogen-dependent. In addition, in contrast to the high affinity of EGF and TGF-α (with K${}_{D}$ of about $10^{-8}$–$10^{-11}$ M), the affinity of AREG for the receptor is significantly lower [38].

According to the established concepts that mostly summarize the data obtained on cell lines of the HeLa type, binding of a ligand to EGFR on the plasma membrane leads to receptors’ dimerization and activation of their TK-domains. Further, according to the cross-mechanism, tyrosine residues on the C-terminal cytoplasmic domain of the receptors are phosphorylated, thus creating binding sites for a number of proteins that initiate signaling cascades, such as MAP- and PI3-kinase, PLC-γ, STAT cascades, etc. Then, RIN1, GEF for the small GTPase Rab5, which is a key regulator of early endosomes, and the c-Cbl, the ubiquitin ligase responsible for sorting the receptor into the lysosomal degradation pathway, also bind to the receptor [39,40]. Both of these proteins are activated by EGFR tyrosine kinase, and EGFR internalization into endosomes and subsequent degradation in lysosomes lead to the so-called downregulation of the receptor, i.e., a decrease in its amount on the plasma membrane, resulting in desensitization of the cell to the action of growth factors and termination of the EGF-produced signal. Differences in the pH dependence of the dissociation of ligand–receptor complexes results in EGF-EGFR complex delivery to lysosomes, where pH is known to be lower then 5.0, while TGF-α-EGFR dissociates in early endosomes at pH 6.0. This dissociation results in EGFR inactivation and recycling back to the plasma membrane without degradation in the lysosomes, thus retaining the ability of repeated cycles of internalization [41,42]. The same recycling route is assumed for AREG [42]. Obviously, the duration and intensity of the signals generated by the EGFR will differ depending on the bound ligand.

However, at present, the exact molecular mechanisms that underlie the difference in EGFR functioning in transformed cells and cultivated enMSCs remain unclear. To come closer to this issue, we have addressed the physiological responses to receptor activation by three different ligands, known to be expressed in endometrium, on several MSC cell lines and on human cervical carcinoma of HeLa line in order to point out “divergence sites” in EGFR system action for further analysis. Although regulation of isolated enMSC in culture, which is a reduced system compared with tissue, is obviously more simplified and cannot be considered as fully identical to physiological ones (and this is true for all cultivated cell lines obtained from multicellular organisms), cell lines have some great advantages, such as ease of obtaining large masses of cells and genetic manipulations with them, as well as the possibility to examine the role of certain compounds or their controlled combinations. Stromal/stem enMSC are easy to acquire by noninvasive procedure from menstrual blood. Despite the above-mentioned features, it can be assumed that the major basic properties and their regulation characteristics for tissue are preserved in isolated cells. Indeed, enMSC lines have high proliferative potential, depend on hormones and growth factors and can be stimulated for tissue-specific differentiation. So, in some sense, they may be a model of normal cells.

In the present paper, we compare some aspects of EGFR functioning in enMSC as an example of normal cells and in tumor-derived HeLa cells. We show that enMCS, as well as some other MSC lines of another tissue origin, express EGF receptors at a high level comparable to that in transformed HeLa cells, while maintaining all signs of normality. We analyzed the effect of three EGF receptor-specific ligands, EGF, TGF-α and AREG, and demonstrated that their effects on proliferation and EGFR degradation differ from those on HeLa cells. Unlike HeLa, EGF and TGF-α, in an EGFR-dependent manner, maintain proliferation in enMSC. We found that in both cell types, prolonged incubation with EGF and TGF-α resulted in EGFR protein degradation, but on the contrary to HeLa, degradation was delayed in enMSC, and EGFR level stayed low, at least for 5 days. We also demonstrated that EGF and TGF-α inhibited stimulated decidualization in cultured enMSC. Our data allow to propose that endosomal regulation can be responsible in part for found differences in EGFR ligand action on the two cell types.

## 2. Results

### 2.1. Estimation of the EGFR Protein Level in Different Mesenchymal Stromal and Transformed Cell Lines

**Figure 1:**
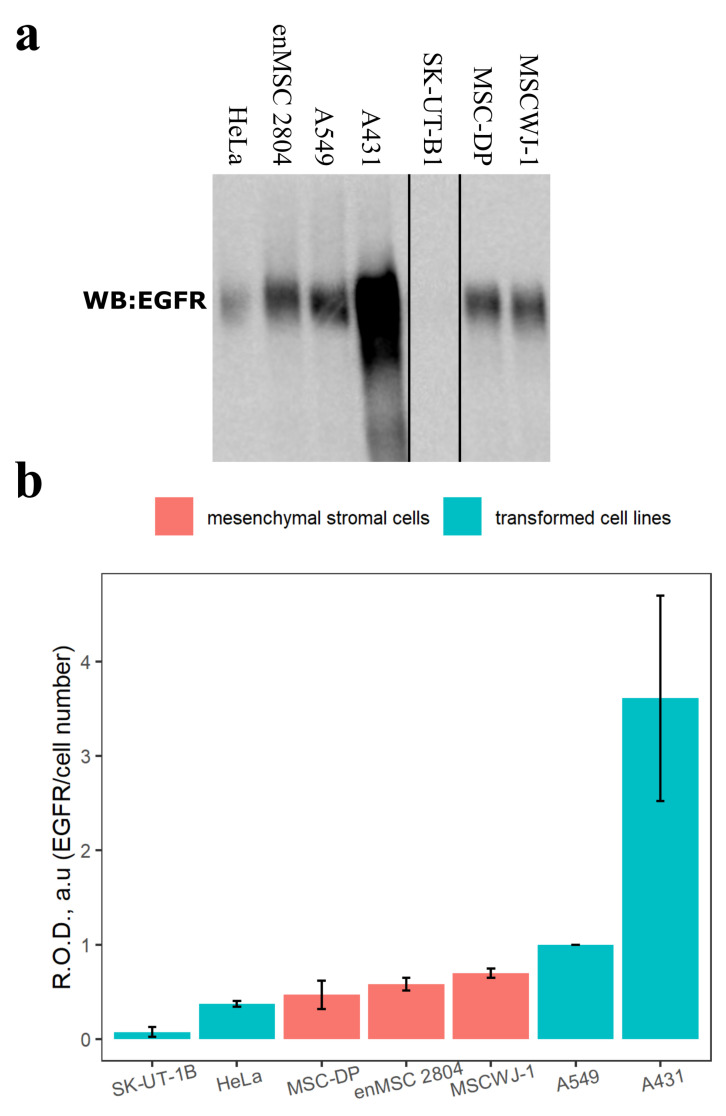
Epidermal growth factor receptor (EGFR) protein level in various mesenchymal stromal and transformed cell lines. Total cell lysates of several mesenchymal stromal cell lines (orange) and transformed cell lines (cyan) were analyzed. Mesenchymal stromal cell lines used: human dental pulp mesenchymal stromal cells (MSC-DP), endometrial mesenchymal stromal cells (enMSC 2804) and human umbilical cord mesenchymal stromal cells (MSCWJ-1). Transformed cell lines used: human uterine leiomyosarcoma cell line (SK-UT-1B), human cervix carcinoma (HeLa), adenocarcinomic human alveolar basal epithelial cells (A549) and squamous cell carcinoma of the vulva (A431). (**a**) EGFR protein level in these cells was examined by SDS-PAGE and Western blotting. Western blots were stained with polyclonal antibodies raised against EGFR (about 170 kDa). Total cell lysate samples were normalized to cell number. (**b**) Relative optical densities (R.O.D., *y*-axis) of EGFR band normalized per cell number are presented in increasing order as the mean ± s.e. of three independent experiments.

First, we estimated the EGFR protein amount in total cell lysates of several mesenchymal stromal cell lines and compared it with the amount of EGFR in transformed cells by SDS-PAGE and Western blotting. As can be seen in Figure 1, the EGFR protein levels per cell in MSC used for analysis (human dental pulp mesenchymal stromal cells (MSC-DP), endometrial mesenchymal stromal cell line (enMSC 2804) and human umbilical cord mesenchymal stromal cells (MSCWJ-1)) are similar to those of transformed HeLa and A549 cell lines and only less than in A431 cell line. All these transformed cells overexpress EGFR, especially A431 [43]. Such significant levels of EGFR in MSCs imply that EGFR plays a considerable role in MCSs homeostasis, although it does not cause MSC transformation. Interestingly, in the transformed cells of human leiomyosarcoma SK-UT-1B originated from placenta and uterus [44], EGFR protein level is several fold lower than in all other MSC and transformed cell lines used.

### 2.2. EGF, TGF-α and AREG Differentially Affect HeLa and enMSC Cell Proliferation

**Figure 2:**
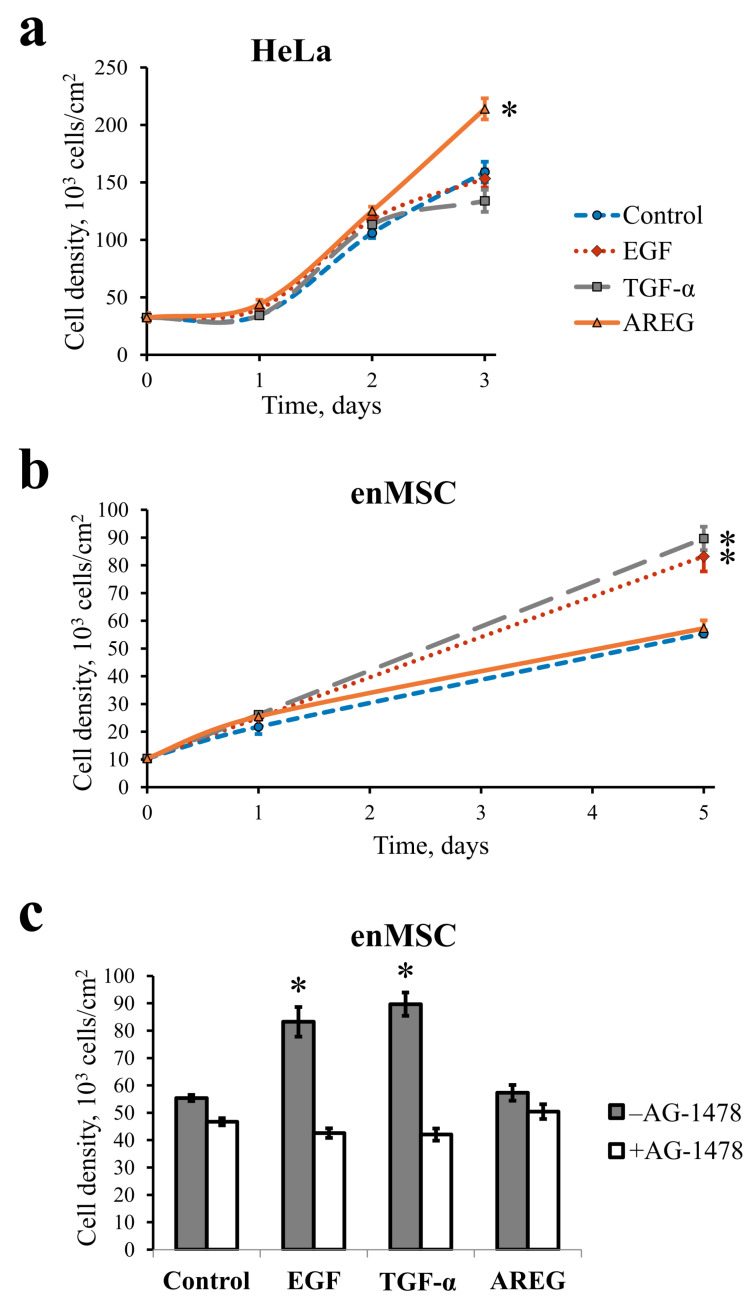
Epidermal growth factor (EGF), transforming growth factor-α (TGF-α) and amphiregulin (AREG) have different effects on cell proliferation of HeLa (**a**) and enMSCs (**b**) cells. EGF (10 nM), TGF-α (20 nM) and AREG (20 nM) were added to HeLa (**a**) or enMSCs (**b**) at day 0, and cells were incubated in this ligands-containing media or identical media without ligands (control) for 3–5 days. Growth curves of HeLa (**a**) or enMSCs (**b**) are presented. (**c**) Pro-proliferative effect of EGF and TGF-α in enMSCs is EGFR tyrosine kinase-dependent. Selective EGFR tyrosine kinase inhibitor Tyrphostin AG-1478 (1 μM, + AG-1478) was added to enMSCs 30 min before EGF, TGF-α or AREG addition. Cell density was estimated at day 5 after AG-1478 and ligands treatment. Data represent the mean ± s.e. of three independent experiments. * *p*-values < 0.05 represent the statistically significant differences.

To examine the effect of three different EGFR ligands—EGF, TGF-α and AREG—on enMSCs and HeLa cell proliferation, we provided the proliferation assay. Briefly, cells were plated at cell density $10^{4}$ cells per cm${}^{2}$. The next day, the medium was replaced with a complete medium containing 10 nM EGF, 20 nM TGF-α or 20 nM AREG. The cell number was calculated using a flow cytometer after 1, 2 and 3 days for HeLa (Figure 2a) and after 1 and 5 days for enMSCs (line 2804) (Figure 2b). We did not include the 5-day point into the analysis for HeLa cells, as by day 3 the cells reached confluency due to the shorter cell cycle compared with enMSC.

As expected, we found that under these experimental conditions, EGF or TGF-α did not influence HeLa cell proliferation, as there were no differences in cell densities up to the 3-day point between the control untreated cells and the cells treated with EGF or TGF-α. On the contrary, AREG addition increased HeLa cell density at day 3 (Figure 2a).

However, in enMSCs, both EGF and TGF-α increased cell proliferation, but no difference in cell density between control and AREG-treated enMSCs was observed at day 5 (Figure 2b). To check whether the proliferative effect of EGF and TGF-α in enMSC is EGFR-dependent, we also treated the cells with EGFR tyrosine kinase inhibitor AG-1478 together with ligands and compared cell density after 5 days. Indeed, we showed that AG-1478 treatment canceled EGF and TGF-α-dependent increase in enMSCs proliferation (Figure 2c).

### 2.3. EGF and TGF-α Suppress Decidualization of Human enMSCs

Next, we evaluated the effect of three EGFR ligands, EGF, TGF-α and AREG, on the tissue-specific differentiation of human enMSCs. The enMSCs of line 2804 were stimulated to decidualization by incubation of cells in differentiation medium based on phenol red-free DMEM/F12 and containing 8-bromoadenosine-3${}^{\prime }$,5${}^{\prime }$-cyclic monophosphate (8-Br-cAMP) and progesterone (P4). EGF (10 nM), TGF-α (20 nM) or AREG (20 nM) were also added to the differentiation medium where indicated, and the cell morphology, as well as IGFBP1, a known secreted marker of decidualization, were analyzed at day 7 after medium addition.

**Figure 3:**
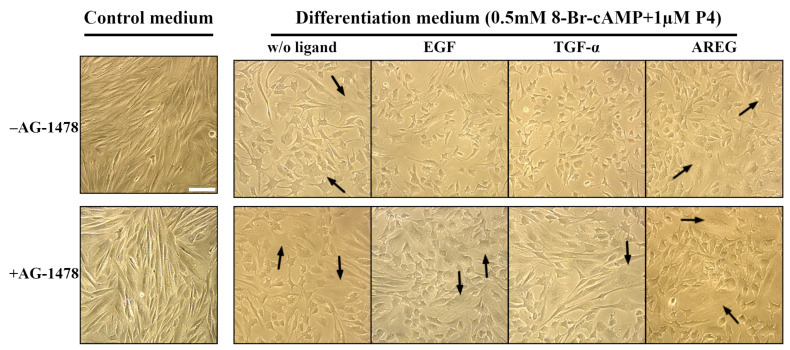
EGF and TGF-α prevent morphological changes typical for decidual differentiation. Decidualization was induced by incubation of enMSC in differentiation medium (phenol red-free DMEM/F12 supplemented with 2% fetal bovine serum and containing 0.5 mM 8-bromoadenosine-3${}^{\prime }$,5${}^{\prime }$-cyclic monophosphate (8-Br-cAMP) and 1 μM progesterone (P4)). EGF (10 nM), TGF-α (20 nM), AREG (20 nM) or no ligand (w/o ligand) were added to differentiation medium. The experiment was carried out in the absence (**upper panel**, −AG-1478) and in the presence (**lower panel**, + AG-1478) of selective EGFR tyrosine kinase inhibitor Tyrphostin AG-1478 (1 μM). Cells were incubated in differentiation medium for 7 days, then their morphology was observed by phase-contrast microscopy. Cells with decidual-like morphology are indicated by the arrows.

The cells, incubated in control medium without decidualization factors 8-Br-cAMP and P4, had fibroblast-like morphology typical for self-renewal phase, i.e., their morphology was not changed during 7 days of experiment (Figure 3). Using ELISA, we detected no IGFBP1 in the medium, as expected (Table 1). But when enMSCs were incubated in differentiation medium for the same time, their morphology was changed. Namely, some number of large cells of polygonal form with large round nuclei appeared (Figure 3). Such morphology was described before as mature decidual cell-like [45,46,47]. ELISA assay verified the morphology analysis: IGFBP1 was detected in the medium (Table 1). This indicates IGFBP1 secretion by the cells and confirms successful decidualization process, as IGFBP1 is the factor secreted by mature decidual cells.

On the contrary, when the cells were incubated in differentiation medium, containing EGF or TGF-α, no cells with decidual-like morphology could be found. Instead, cells looked smaller than undifferentiated and decidualized ones. No IGFBP1 secretion was detected there, either (Table 1). At the same time, AREG addition did not prevent enMSCs decidualization, as many cells incubated in AREG-containing differentiation medium showed decidual-like morphology (Figure 3). According to that, ELISA assay also confirmed the presence of a detectable amount of IGFBP1 in the medium of cells stimulated with AREG (Table 1).

Tyrphostin AG-1478 abolished the inhibitory effect of the two ligands on the decidual differentiation of eMSCs, which was confirmed by both morphological and ELISA IGFBP1 secretion analysis. Namely, in the wells with differentiation medium without ligand or with EGF/TGF-α/AREG in presence of AG-1478, we found large cells with decidual-like morphology. According to ELISA analysis, in all wells with AG-1478-containing differentiation medium, a high level (>16,000 pg/mL) of IGFBP1 secretion was found (Table 1). This indicates that EGF- and TGF-α-dependent ablation of enMSCs decidual differentiation is EGFR-mediated.

### 2.4. AREG Is Secreted by Human enMSCs

As AREG, contrary to EGF and TGF-α, has no influence on enMSCs proliferation and differentiation, we supposed that these cells may secrete AREG that results is an autocrine loop, which mimics the effect of exogenously added ligands. To check this, we analyzed the presence of AREG in the conditioned medium of two different human endometrial MSCs lines (enMSC 2804, enMSC 2602), as well as of human dental pulp mesenchymal stromal cells (MSC-DP), human umbilical cord mesenchymal stromal cells (MSCWJ-1) and human-placenta-derived mesenchymal stromal cells (MSC-PL2). We also used the human breast adenocarcinoma MCF-7 cell line as the positive control, as MCF-7 cells are known to secrete AREG [48]. Cells were grown to confluence, and then the growth medium was changed and collected for ELISA analysis 2 days later.

We detected AREG in the conditioned medium of all enMSC lines, MSC-PL2 and MCF-7 cells (Table 2). On the contrary, no detectable AREG levels were identified in the conditioned medium of the MSC-DP, MSCWJ-1 and HeLa cells (Table 2). Note that the AREG secretion was detected in cells derived from estrogen-dependent tissues, i.e., endometrium and placenta, as well as in the malignant MCF-7 cell line, which is also estrogen-dependent.

### 2.5. EGFR Endocytosis Is Stimulated by EGF and TGF-α but Not by AREG in Both enMSCs and HeLa Cells

It is believed that the binding of a ligand to EGFR results in fast internalization of ligand–receptor complexes into early endosomes, while the choice of recycling or degradation pathway is determined later at the endosomal level. So, we compared the behavior of EGFR after addition of the three ligands with enMSCs and HeLa cells. EGF- and TGF-α-induced EGFR endocytosis was stimulated in these cells according to pulse-chase protocol, i.e., EGF (10 nM) or TGF-α (20 nM) was added to cells for 5 min (pulse) followed by the washout of unbound ligands and chase period at 37 °C. This protocol allows to follow the synchronous wave of endocytic events while avoiding possible distortions associated with recycling or saturation of the endocytic apparatus. Cells were fixed and immunofluorescently labeled with anti-EGFR antibodies, and the EGFR intracellular distribution was analyzed using confocal microscopy.

**Figure 4:**
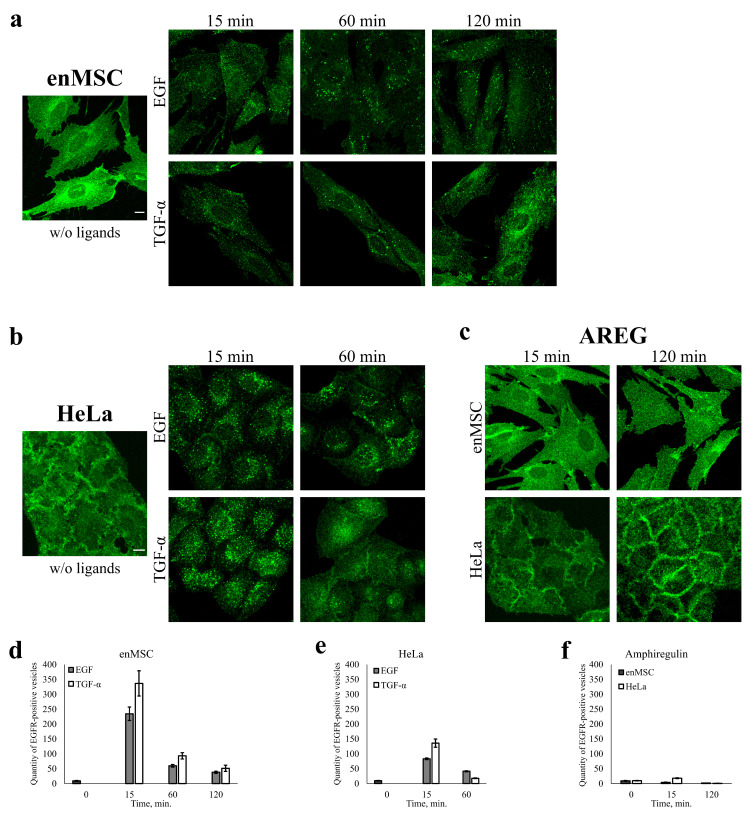
EGFR endocytosis is stimulated in human enMSCs and HeLa cells by EGF and TGF-α but not AREG addition. EGF- and TGF-α-induced EGFR endocytosis was stimulated in enMSCs (**a**,**d**) and HeLa cells (**b**,**e**) according to pulse-chase protocol: EGF (10 nM) or TGF-α (20 nM) was added to cells for 5 min (pulse) followed by washout of unbound ligand and chase period at 37 °C. AREG (20 nM) was added to enMSCs (**c**, upper panel) and HeLa cells (**c**, lower panel) without subsequent washout. Cells, treated with ligands or not (w/o ligands), were fixed at the time indicated (15, 60 and 120 min) and immunostained using antibodies against EGFR. Maximum intensity projections of the typical cells (**a**–**c**) are presented (scale bar—10 μm). The number of EGFR-positive vesicles after EGF (**d**), TGF-α (**e**) or AREG (**f**) addition was quantified.

In control cells (not stimulated with any ligand), we observed predominantly plasma membrane EGFR distribution with no or a few dim EGFR-positive vesicles in the cytoplasm (Figure 4). Upon EGF stimulation, EGFR is internalized both in HeLa and enMSCs, as several dozens of EGFR-containing endosomes could be seen in the cytoplasm of the cells 15 min after ligand addition (Figure 4). In full accordance with numerous data for HeLa cells, including our own [49], endosomes with EGF enlarge and show mostly juxtanuclear localization by 60 min, indicating endosomal maturation and delivery to lysosomes. Upon TGF-α stimulation, a lot of EGFR-positive vesicles are also detected inside HeLa cells at 15 min; however, after 60 min, mostly plasma membrane staining is detected, which is typical for well-established recycling pathways of EGFR after stimulation with TGF-α in HeLa (Figure 4b).

As seen in Figure 4, enMSC cells actively internalize the EGFR receptor bound both to EGF and TGF-α, but EGFR-positive vesicular staining persists for 120 min after endocytosis stimulation without pronounced enlargement of endosomes and their redistribution to the juxtanuclear region of the cells (Figure 4a).

It is important to emphasize that both ligands behave in the same way in MSCs, in contrast to HeLa. Also, while in HeLa EGFR is localized mainly on the basolateral surface, in MSCs, it is more likely to be distributed apically; therefore, the contours of these cells are not visible on micrographs as clearly as in the control HeLa. This fact, as well as the strong spreading of enMSCs, does not allow us to assert that they are completely unable to recycle some portion of TGF-α and EGF; however, it is obvious that this pathway is not dominant, as it is for TGF-α in HeLa. To sum up, we showed that EGFR is internalized upon EGF and TGF-α addition both in HeLa and enMSCs, but the intracellular fate and endocytosis dynamics of both ligands are different in enMSC and HeLa.

It was reported earlier that AREG induces EGFR recycling [42], that is, it behaves like TGF-α in HeLa. In the above-mentioned experiments, we used a short pulse to initiate internalization of ligand–EGFR complexes. However, with this protocol, no EGFR internalization upon AREG addition was detected.

We propose that one of the reasons may be lower affinity of AREG to EGFR; so, to give AREG enough time to form stable complexes with EGFR, AREG was added to the cells without subsequent washout. Despite this, upon AREG addition, no EGFR-positive vesicles were detected both in enMSCs and HeLa cells up to 120 min point. During this period, the localization of EGFR was typical for plasma membrane in both cell types: lateral for HeLa and apical for enMSC (Figure 4c).

### 2.6. EnMSC Incubation with EGF Leads to a Long-Term Decrease in the Level of EGFR

**Figure 5:**
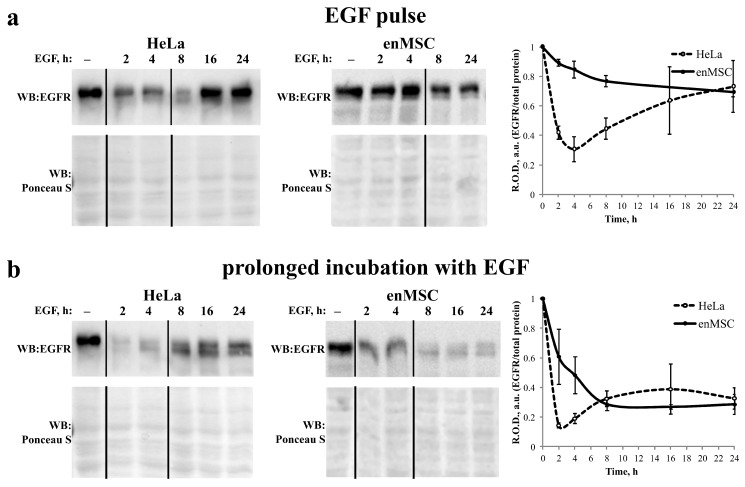
EGFR degradation is slowed down in enMSCs compared with HeLa cells. The EGFR endocytosis was stimulated in enMSCs and HeLa cells by the addition of 10 nM EGF according to (**a**) pulse-chase protocol (ligand was added to the cells for 5 min with subsequent washout from the unbound ligand and chased for the times indicated) or (**b**) prolonged incubation with EGF (ligand was present in the medium for the whole time of the experiment). At 2, 4, 8, 16 and 24 h, the cells were processed for Western blotting. The blots were stained with polyclonal antibodies raised against EGFR. Ponceau S staining was used as the loading control. Representative blots are presented. Graphs in the right panels show quantification of EGFR level based on EGFR band densitometry. Relative optical densities (R.O.D., *y*-axis) were normalized for the density of EGFR band in control cells before EGF addition. Data represent the mean ± s.e. for 3 independent experiments.

We demonstrated that the dynamics of EGF-induced EGFR endocytosis seems to be significantly slowed down in enMSCs compared with HeLa cells and proposed that there is a difference in the rate of EGFR degradation in the two cell types. To check this, we stimulated EGFR endocytosis by the addition of EGF in a close-to-saturating concentration (10 nM) according to two different protocols. In the pulse-chase protocol, EGF was added to the cells for 5 min; then, the cells were washed out from the unbound ligand and chased for 2–24 h. In this case, a relatively synchronous wave of endocytic events was stimulated, but it did not involve all surface EGF receptors (Figure 5a). In the prolonged incubation protocol, EGF at the same concentration was added to the cells without subsequent washout for all 24 h of the experiment (Figure 5b). When using the second protocol, recycled (if any) and synthesized de novo EGF receptors should eventually be internalized and delivered to lysosomes for degradation due to the long-term presence of the ligand in the medium. At the time indicated, total cell lysates were prepared and subjected to SDS-PAGE and Western blot analysis with anti-EGFR antibodies. Ponceau S staining was used as the loading control.

As one can see in Figure 5, in HeLa cells, the level of EGFR protein decreased for 60–80% in the pulse-chase experiment and by about 90% with the permanent presence of EGF within 2–4 h. Then, the amount of EGFR protein recovered, and this restoration was more pronounced when the pulse-chase protocol was used, while at prolonged incubation protocol it is not significant. These data are consistent with the generally accepted hypothesis that EGFR degradation stimulates its de novo synthesis, which restores the sensitivity of cells to growth factors. However, we found that it is not true for enMSCs. Upon EGFR endocytosis stimulation, the amount of EGFR was decreasing gradually and much more slowly than in HeLa cells, even at permanent EGF presence in the incubation medium. In this case, it reached a minimum of about 20% of initial value within 8 h and then practically did not change. No restoration was detected up to 24 h. In pulse-chase experiments, EGFR level decreased by not more than 30% of the initial value by 24 h.

**Figure 6:**
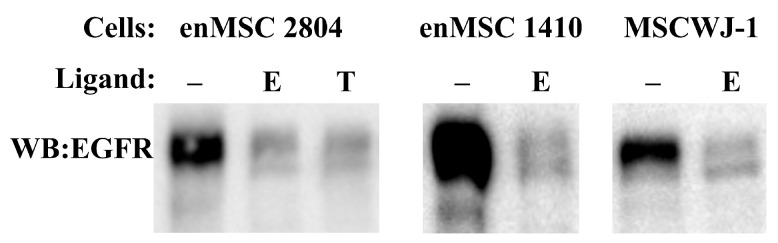
EGFR amount remains low in MSCs at the 5th day of permanent EGFR ligand treatment. An amount of 10 nM EGF (E) and 20 nM TGF-α (T) were added to enMSC 2804, enMSC 1410 and MSCWJ-1 cells in the DMEM/F12 medium, supplemented with 10% fetal bovine serum. Cells were incubated in ligand-containing media for 5 days. Total cell lysates were normalized to cell number. The extent of EGFR degradation in these cells was analyzed by SDS-PAGE and Western blotting with polyclonal antibodies raised against EGFR.

It may be proposed that not only degradation but also EGFR level restoration is slower in enMSC than in HeLa cells. To check this, we compared the level of EGFR protein in two enMSC cell lines and MSC from Warton jelly (MCSWJ-1) after 5 days incubation with ligands (Figure 6). In all three cases, EGFR level was low and comparable to the level detected in experiment presented in Figure 5. The same was found for enMSC 2804 incubated with TGF-α. This indicates that the both ligands stimulate EGFR degradation in a similar manner. So, we conclude that in contrast to HeLa, either EGF or TGF-α downregulate EGFR but do not stimulate essential receptor synthesis de novo, even for a very long time.

**Figure 7:**
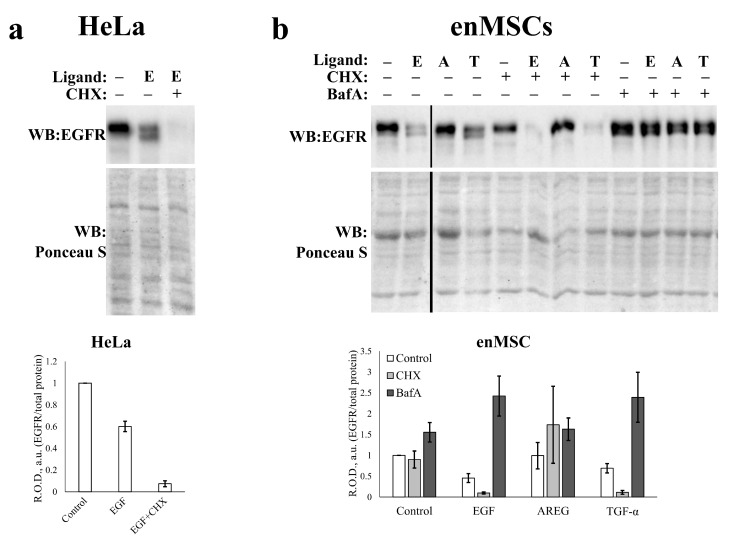
Estimation of EGFR degradation 24 h after EGFR ligands treatment of the enMSCs and HeLa cells. The EGFR endocytosis was stimulated in HeLa cells (**a**) and enMSCs (**b**) by the addition of 10 nM EGF (E), 20 nM TGF-α (T) or 20 nM AREG (A) according to prolonged ligand incubation protocol. The extent of EGFR degradation in these cells was analyzed by SDS-PAGE and Western blotting with polyclonal antibodies raised against EGFR. Ponceau S staining was used as the loading control. Inhibitor of eukaryotic translation cycloheximide (35.5 μM, CHX) and vacuolar V-ATPase inhibitor bafilomycin A${}_{1}$ (100 nM, BafA) was added to the cells 30 min before and then simultaneously with ligand addition. The equal amount of DMSO was added to cells not treated with the inhibitors. Graphs shows quantification of EGFR level based on EGFR band densitometry. Ponceau S staining was used as the loading control. Relative optical densities (R.O.D., *y*-axis) were normalized for the density of EGFR band in control cells before EGF addition. Data represent the mean ± s.e. for 3 independent experiments.

To estimate directly the level of EGFR de novo synthesis, we incubated the cells with EGFR ligands in the presence of the inhibitor of eukaryotic translation cycloheximide (CHX) for 24 h. The data presented in Figure 7 demonstrate that CHX practically completely prevents EGFR level restoration in HeLa cells incubated with EGF (Figure 7a), which means a high level of receptor de novo synthesis in response to EGF treatment. In enMSC 2804, incubation with both EGF and TGF-α resulted in EGFR degradation by 24 h, similar to the data in Figure 5. We revealed that the extent of EGFR degradation in EGF- and TGF-α-stimulated enMSCs, treated with CHX, is also more pronounced than in not-treated cells (Figure 7b). This indicates that EGFR synthesis de novo takes place simultaneously with the receptor degradation and that almost all the EGFR is degraded upon EGF- and TGF-α-treatment in enMSC as well as in HeLa. This means that the difference in the receptor dynamics degradation in these two cell types can hardly be explained at the level of differences in the regulation of the receptor protein translation.

As the endocytosis process is accompanied by the endolysosomal acidification, and the acidification of lysosomes is necessary for the cargo degradation [50], we checked if the blockage of endolysosomal acidification would affect the EGFR degradation in enMSC. For this purpose, we used vacuolar V-ATPase inhibitor Bafilomycin A${}_{1}$ (100 nM), which was also added to the cells 30 min before and then simultaneously with ligand addition. Our results prove that EGFR degradation stimulated by both EGF and TGF-α was ablated by Bafilomycin A${}_{1}$ treatment (Figure 7b). We conclude that EGFR undergoes degradation within the acidic endolysosomal compartments in enMSC as well as in HeLa cells. AREG was a kind of positive control in this experiment: as a noninternalized ligand, it also does not stimulate EGFR degradation or its de novo synthesis.

## 3. Discussion

In this study, we tried to characterize the role of EGFR and its homeostasis in cultured endometrial mesenchymal stromal cells isolated from menstrual blood in comparison with tumor-derived HeLa cells. EnMSCs are highly proliferative when growing in medium supplemented with serum and can be stimulated for tissue-specific differentiation, known as decidualization. Thus, being isolated from both the influence of their niche in the endometrium and the hormonal regulation of the organism, cultured cells retain tissue-specific features. Although it was known that EGF may modulate proliferation and differentiation of MSCs [51,52], we, for the first time to our current knowledge, demonstrated that EGFR is expressed in several proliferating MSC lines of different origin at the level compared with cancer cell lines known to overexpress EGFR, such as HeLa and A549. As widely recognized, malignization is often associated with EGFR overexpression, as well as with the accumulation of EGFR-related mutations, which lead to the deregulation of EGFR-mediated signaling. These changes are often accompanied by increased EGFR ligand production due to autocrine loops [24,29,31,32]. According to our expectations, both EGF and TGF-α in complete serum-supplemented medium did not produce any additional effect on proliferation rate compared with complete medium alone. However, we showed that in contrast to tumor-derived HeLa cells, human enMSC retain their proliferation dependence on two EGFR ligands, EGF and TGF-α (Figure 2). This indicates that a large number of EGFR receptors (about few hundreds of thousands per cell) is not necessarily associated with transformation. Interestingly, in early work by Niikura et al., it was reported that immunostaining of EGFR was found for 58% of normal endometrial biopsies, 100% of endometrial hyperplasia and about 67% of endometrial carcinoma specimens, which most probably indicates that both normal and abnormal endometria are heterogeneous in terms of EGFR expression [53]. However, the latter may also be due to the ability of cells in endometrium, but not isolated cells, to cyclically alter (though not fully synchronously) expression levels of EGFR family receptors and their ligands during the menstrual cycle [9,14].

On the contrary, the third ligand, AREG, demonstrated pro-proliferative activity on HeLa but not on enMSC cells. These differences may have at least two explanations: First, we also found that AREG does not stimulate any detectable EGFR internalization, and earlier, it was believed that mitogenic signal from EGFR is generated at the plasma membrane but not in endosomes [54]. However, this idea should be abandoned, because AREG shows opposite effects on proliferation of HeLa compared with enMSC but does not undergo internalization in both cell lines. On the other hand, we also showed the significant level of soluble AREG secretion by all tested enMSC lines but not by HeLa cells (Table 2). So, more probably, enMSC just does not sense exogenously added AREG, as the endogenous AREG is already binded to most of the receptors on the cell surface. This suggestion is not in conflict with the effects of exogenously added EGF and TGF-a on enMSC due to much higher affinity for the receptor of the latter factors [55]. Conversely, HeLa cells are known to release low levels of EGF precursor [56] and that may be the reason for their insensitivity to the exogenous EGF in the proliferation assay.

The next important point concerns the effect of EGFR ligands on enMSC decidualization. As was mentioned above, enMSC cells preserve their typical features in culture, such as the ability to decidualize. EGFR was earlier demonstrated to be involved in this process [6,17]. We found that enMSC stimulated by the addition of 8-Br-cAMP and P4 successfully enter differentiation program, which is indicated by secreting IGFBP1, while the addition of EGF and TGF-α, but not AREG, to stimulated cells inhibited morphological changes and IGFBP1 production. Our data are in line with some publications [52] but in contradiction with several other studies [6]. The main concern is the stage of the menstrual cycle at which EGFR operates as the regulator of differentiation—a proliferative or secretory one. For example, in some works, the highest EGFR mRNA expression was found in the early proliferative phase [9], while the others have demonstrated that EGFR mRNA levels are significantly higher at the secretory stages than at the early follicular stage [14,15].

However, two suggestions can be presented. First, its again important to stress that many studies were carried out on endometrial biopsy material, which obviously represent more physiological and more complicated systems than the cells in culture. In endometrium, EGFR was found in the endometrial epithelium [9,14,57] and endometrial stroma [57]. Secretion of EGFR ligands EGF, TGF-α and AREG, as well as HB-EGF and betacellulin, was also detected in the endometrium, [9,14]. Also, EGFR and its ligands’ levels were reported to change through the menstrual cycle [9,57] under estrogen/progesterone control. Importantly, however, ligands can be synthesized by different types of cells that form the endometrium and cannot be attributed specifically to MSCs. So, many effects may be mediated by juxta-, para- and endocrine modes. In the present work, we examined the role of EGFR and its ligands in reduced system of enMSCs in culture. Isolated enMSC cells may be called a self-regulating system only within themselves and the sets of receptors and ligands they synthesize. Today, our understanding of the extent to which the endometrium as a tissue and the lines derived from it are equivalent is grossly incomplete.

We reported here that enMSC incubation in the presence of EGF and TGF-α resulted in slow receptor degradation and its stabilization at very low levels for at least 5 days, while in HeLa, these factors stimulate fast degradation and restoration of initial levels in 24 h. At first glance, such dynamics in enMSC contradicts the role of the receptor in decidualization. However, firstly, a low level of the receptor does not indicate its absence, especially since we showed that a level of de novo receptor synthesis is comparable to that in HeLa (Figure 7), which makes it possible for the receptor to be fully involved in signaling. Second, the menstrual cycle is a system of well-orchestrated successive events. It was reported that successful decidualization occurs through two steps and needs the proliferative wave for initiation of the differentiation program [6]. If EGFR synthesis in endometrium is under control of estrogen, it also possible that the late wave of this hormone can raise EGFR content at the secretory phase. Under our conditions, enMSCs grew and were stimulated in the absence of estrogen, which may explain the inhibitory effect of EGF on decidualization in cultured cells. Undoubtedly, this issue has to be addressed in future experiments. Importantly, in the presence of EGFR TK inhibitor AG-1478, the level of IGFBP1 was significantly higher than in the presence of EGF and TGF-α alone, which means that other EGFR ligands than were used in our experiments also activate EGFR in culture, and they also make a significant contribution. Indeed, the role of HB-EGF was also reported [9,15].

One of the main points of the present work is the finding of significant differences in the intracellular behavior of EGFR bound to the three ligands in enMSC that do not fit with the current paradigm, which is presented by Hela cells. The latter supposes that all the ligands induce EGFR internalization, but due to different sensitivity of ligand dissociation to intraendosomal pH level, EGF targets EGFR for lysosomal degradation, while TGF-α and AREG promote EGFR recycling [42]. This means that only EGF should actively stimulate degradation of EGFR protein. This view has been proved in Hela cells for EGF and TGF-α by (i) confocal microscopy that demonstrates typical behavior of EGF-EGFR-containing endosomes that due to effective fusions of early endosomes, became enlarged with time and concentrated in the juxtanuclear region of the cells, where lysosomes are localized, and (ii) by Western blotting, which shows a decrease in total EGFR protein within 2 h. At the same time, TGF-α produce numerous endosomes, but in 60 min, TGF-α–stimulated EGFR was detected, mostly in faintly stained recycling structures and on plasma membranes, which indicates effective recycling. On the contrary, in enMSCs, both the ligands formed endosomes that persisted in cytoplasm for at least 2 h and then caused a decrease in EGFR, thus confirming the protein degradation. The acidification dependence of EGFR degradation indicates involvement of lysosomes in both cell types. Summarizing, the most prominent difference between HeLa and enMSC is that in the latter cells (i) both the ligands behave similarly and (ii) EGFR degradation progresses much more slowly than in HeLa. The third important finding is that in HeLa cells, fast downregulated EGFR returns to its initial level in 24 h (the effect is more pronounced in the pulse-chase experiment, when short addition of the ligand stimulated only one cycle of internalization–degradation in opposition to the constant ligand presence in the medium), while in enMSC, EGFR stayed at the minimal (but detectable) level for at least 5 days of the experiment. This phenomenon may reflect different mechanisms of EGF endocytosis in enMSC and HeLa. Indeed, in our preliminary experiments, we found that in enMSC, only a small portion of internalized EGFR colocalized with EEA1 early endosomes, which indicates ineffective fusions of EGFR-containing endosomes and, possibly, their delayed maturation into late endosomes or employment of an other than canonical endolysosomal degradation pathway. Anyway, our data undoubtedly indicate that the endosomal stage of the receptor life cycle is involved in the regulation of its functions in enMSCs, though this field is not intensively studied.

Interestingly, AREG did not induce EGFR degradation, and it also hardly stimulated EGFR internalization in the both HeLa and enMSC cells, as no vesicular EGFR-positive endosome-like structures were detected at times typical for the recycling pathways. During the whole time of the experiment, EGFR was localized on a plasma membrane.

What can be the mechanisms underlying these specific effects for both the cell lines and ligands? As for ligands, it is known that they can produce different profiles of EGFR tyrosine phosphorylation in regard to the both phosphorylation sites and the extent and duration of their modification. For example, phosphorylation of Tyr-1045, the binding site of ubiquitin ligase c-Cbl responsible for targeting lysosomal degradation, was reported to be effective by EGF but not by AREG [58]. Moreover, EGFR can form dimers with other members of c-Erb B family receptors, the expression profile of which can depend on the cell line. Also, EGFR may be phosphorylated by serine–threonine kinases [59] and participate in transactivation processes [24]. These variations can also affect the way of internalization [60] and consequently the regulation of intracellular EGFR fate. Finally, we speculate that enMSCs can be more sensitive to autophagy stimulation, and if so, the decrease in EGFR progression on the endolysosomal pathway may occur due to competition of some key regulators of lysosomal and autophagosomal processes [61,62,63].

The obvious reason for maintaining low levels of EGFR protein in enMSCs compared with HeLa might be due to inhibition of the receptor de novo synthesis in these cells; however, our results for CHX effects do not favor this idea. Nevertheless, the dynamics of not only protein levels but also those of mRNA are also necessary to completely answer this question [64].

## 4. Materials and Methods

### 4.1. Cell Cultures

Several human mesenchymal stromal cell (MSC) lines were used. Three lines (2804, 2602 and 1410) of endometrial MSCs were obtained from the MSC collection of the Department of Intracellular Signaling and Transport of the Institute of Cytology of the Russian Academy of Sciences. These cell lines were derived from desquamated endometrium from menstrual blood samples and characterized previously [65]. The human dental pulp MSC (MSC-DP), human umbilical cord MSC (MSCWJ-1) and the human-placenta-derived MSC (MSC-PL2) were obtained from the Center for Shared Use “Collection of Vertebrate Cell Cultures”, supported by a grant from the Ministry of Science and Higher Education of the Russian Federation (Agreement #075-15-2021-683, Institute of Cytology RAS, St. Petersburg). MSC lines were used at passages 5-17 since primary isolation. MSCs were maintained in complete medium based on DMEM/F12 with phenol red (Gibco, Paisley, UK), containing 10% fetal bovine serum (Biowest, Nuaillé, France), GlutaMAX™ (Gibko, UK) and antibiotic/antimycotic solution (Capricorn, Düsseldorf, Germany).

We also used several human cancer cell lines obtained from Russian Cell Culture Collection (Center for Shared Use “Collection of Vertebrate Cell Cultures”) of the Institute of Cytology RAS, Russia, namely derivatives of human cervix carcinoma HeLa cell line, human breast adenocarcinoma MCF-7, human uterine leiomyosarcoma SK-UT-1B, human lung carcinoma cell line A549 and human epidermoid carcinoma A431 cell line. MCF-7 cells were maintained in DMEM/F12 (Gibco, UK), containing 10% fetal bovine serum (Biowest, France), GlutaMAX™ (Gibko, UK) and antibiotic/antimycotic solution (Capricorn, Germany). Other cancer cell lines were cultured in DMEM medium with glucose concentration 4.5 g/L and phenol red (Gibco, Billings, MT, USA), supplemented with 10% fetal bovine serum, (Biowest, France), GlutaMAX™ (Gibko, UK) and antibiotic/antimycotic solution (Capricorn, Germany).

All used cell lines were maintained at 37 °C in the atmosphere of 5% CO${}_{2}$. For the experiments, cells were seeded with a density of $10^{4}$ cells/cm${}^{2}$ for MSC and $5\times 10^{3}$ cells/cm${}^{2}$ for cancer cell lines. When required, cells were serum-starved in growth medium, containing 1% serum for 12 h before the experiment.

### 4.2. Reagents

EGF was purchased from Invitrogen, Carlsbad, CA, USA. AREG and TGF-α were from R&D Systems, Minneapolis, MN, USA. If not otherwise indicated, 10 nM of EGF and 20 nM AREG or TGF-α were used. EGFR inhibitor AG-1478 (Sigma-Aldrich, Saint Louis, MO, USA) was used at a concentration of 1 μM. The protein synthesis inhibitor cycloheximide (CHX, Sigma-Aldrich, USA) was used at a concentration of 35.5 μM. The vacuolar V-ATPase inhibitor bafilomycin A${}_{1}$ (BafA, Sigma-Aldrich, USA) was used at a concentration of 100 nM.

### 4.3. Proliferation Assay

In order to evaluate the effect of the EGFR ligands on proliferation, we plated as indicated above. Next day, the medium was replaced with complete medium, containing growth factors and inhibitor, as indicated. After the 1st, 2nd, 3d or 5th days, the cells were detached with 0.05% trypsin (Gibko, UK) and counted using flow cytometer CytoFLEX (Beckman Coulter, Brea, CA, USA).

### 4.4. Decidualization Induction

Decidual differentiation of enMSCs was induced by adding phenol red-free DMEM/F12 medium containing 0.5 mM 8-bromoadenosine- 3${}^{\prime }$,5${}^{\prime }$-cyclic monophosphate (8-Br-cAMP, Sigma, Livonia, MI, USA), 2% fetal bovine serum, 1 μM progesterone (P4, Sigma, USA), GlutaMAX™ and antibiotic/antimycotic [66]. The medium was refreshed at the 3d and 6th day. The degree of differentiation was determined by estimation of cell morphology using phase-contrast microscopy (20× objective) and secreted IGFBP-1 amounts using ELISA on the 7th day after induction.

### 4.5. Western Blotting

For the total cell lysate preparation, cells grown on 60 mm dishes (ThermoFisher Scientific, Waltham, MA, USA) were resuspended in 60 μL lysis buffer containing 1% Triton X-100 (Serva Serving Scientists, Heidelberg, Germany), 20 mM Tris-HCl, 150 mM NaCl, 1 mM EGTA, 1 mM EDTA, Halt Protease and Phosphatase Inhibitor cocktail (ThermoFisher Scientific, #78442, USA) and 1 mM Na${}_{3}$VO${}_{4}$. For the lysate normalization for cell number, the cells were counted using flow cytometry (as described in Section 4.3) before pelleting by centrifugation at 1500 rpm for 10 min, and then 40 μL of lysis buffer per $10^{5}$ cells was added to the pellet. Cell lysates were homogenized using a 27 G syringe. The supernatants were collected after centrifugation at 4000 rpm for 5 min at 4 °C and mixed with Laemmli sample buffer, with subsequent incubation for 5 min at 99 °C. Protein concentrations were determined according to Bradford protein assay using Multiskan FC Microplate Photometer (ThermoFisher Scientific, USA). Samples were run on 7.5% polyacrylamide gels according to Laemmli [67]. After SDS-PAGE electrophoresis, the proteins were blotted for 12 min onto a nitrocellulose 0.45 μm membrane (Bio-Rad Laboratories, Hercules, CA, USA) using Pierce™ 1-Step Transfer Buffer(ThermoFisher Scientific, USA) and the semidry transfer unit (ThermoFisher Scientific Pierce Power System #22830, USA). For identifying the proteins transferred onto the nitrocellulose membrane, Ponceau S (Sigma, USA) staining was performed.

Immunoblotting and ECL (ThermoFisher Scientific, USA) detection were performed according to standard manufacturer’s protocols (Bio-Rad Laboratories, USA). Antibodies against EGFR (#4267S, Cell Signaling Technology, Danvers, MA, USA) at 1:1000 dilution and horseradish peroxidase-conjugated goat antirabbit IgG (GAR-HRP, Cell Signaling Technology, USA) at 1:2000 dilution were used. ImageLab 6.0.1 software (Bio-Rad Laboratories, USA) was used for the analysis of Western blotting images.

### 4.6. Immunofluorescence and Laser Scanning Confocal Microscopy

The cells were grown on coverslips up to about 70% monolayer on the day of the experiment. For stimulation of endocytosis according to pulse-chase protocol, the ligand was added to the cells for 5 min (pulse), followed by the washing out of unbound ligands and chased for indicated time. All incubations were carried out at 37 °C. In some cases, ligands were added to the cells and were present in incubation medium during the whole time of the experiment (prolonged incubation). At the indicated time after stimulation, the cells were fixed with 4% formaldehyde for 15 min at room temperature (RT). After that, the cells were washed with PBS and then permeabilized with 0.5% Triton X-100 for 15 min at RT. After washing, the cells were blocked in 1% BSA for 30 min at RT to prevent nonspecific antibody binding and then incubated overnight at 4 °C with polyclonal rabbit antibodies against the intracellular domain of EGFR (#4267S, Cell Signaling Technology, USA), in 1% BSA at 1:100 dilution. After washing with PBS, the cells were incubated with secondary antibodies GAR-Alexa Fluor 647 (ThermoFisher Scientific, USA) at 1:200 dilution for 20 min at 37 °C. Finally, the cells were mounted in 0.2 M DABCO (1,4-diazabicyclo(2.2.2)octane) glycerol-containing media. Distribution of fluorescently labeled proteins in cells was analyzed by confocal laser scanning microscope Olympus FluoView3000 (Olympus, Tokyo, Japan) with 40×/1.3 objective, laser 640 nm. Three to five fields were registered in XYZ projections. Series of optical sections were collected with a 0.5 μm step.

To estimate the number of EGFR-positive vesicles, z-stacks were segmented using Ilastik ver. 1.4.0 software [68]. The segmented vesicles were then counted using 3D Objects Counter plugin [69] for Fiji (ImageJ ver. 1.53 h) [70]. For each time point and control, we analyzed images containing at least 30 cells.

### 4.7. ELISA

The amounts of secreted AREG and IGFBP-1 were quantified in the cell supernatants by Amphiregulin Human ELISA Kit (#DAR00, R&D Systems, USA) and Human IGFBP-1 ELISA Kit (#DGB100, R&D Systems, USA). The data were normalized to the total amount of cells, counted using flow cytometer CytoFLEX (Beckman Coulter, USA). Positive and negative controls provided by the manufacturer were performed in parallel for comparisons. Optical density was obtained using the multifunction analyzer Thermo Scientific Varioskan LUX (ThermoFisher Scientific, USA) at 450 nm. The background was also corrected by measuring the absorbance at 570 nm.

### 4.8. Statistics

All the experiments were repeated at least three times. The data were analyzed using R programming language [71], as well as Microsoft Excel 2007. For the growth curves analysis, type III two-way ANOVA with Tukey’s multiple comparison test was used. A *p*-value <0.05 was considered significant.

## 5. Conclusions

In the present work, we compared the effect of three EGFR ligands with different affinities to the receptor and different intracellular fate in cultivated enMSC and in tumor-derived HeLa cell line. Using several MSC lines of different tissue origin, we found that the level of EGFR in these cells is similar to that in Hela. This means that the overexpression of EGFR is not ultimately bound to malignization, since MSC preserves tight growth and differentiation control. Our findings demonstrated opposing effects of EGF, TGF-α and AREG ligands on proliferation and different dynamics of EGFR degradation and restoration in MSC and HeLa that possibly reflect the difference in regulating intracellular behavior of internalized receptors, indicating the importance of the endosomal stage in cell response.

To realize whether the revealed differences are particular properties of the specific lines used or are based on more general mechanisms that distinguish between normal and transformed proliferating cells, systematic studies are needed on a wider panel of cellular objects. enMSCs attract attention as model objects for the study of various endometrial pathologies and the development of approaches for their treatment. However, at present, there are many contradictions in the data from different research groups obtained from biopsy samples and isolated cell lines. This indicates that a deeper understanding of how the processes in cell lines correspond to those in the whole endometrium is necessary.

## Figures and Tables

**Table 1:** Qualitative estimation of IGFBP1 amount in conditioned medium during decidualization of enMSCs.

	Control			Differentiation (0.5 M 8-Br-cAMP + 1 μM P4)								
	− AG-1478	+ AG-1478		− AG-1478					+ AG-1478			
	w/o lig.	w/o lig.		w/o lig.	EGF	TGF-α	AREG		w/o lig.	EGF	TGF-α	AREG
IGFBP1 secretion	−	−		+	−	−	+		++	++	++	++

EnMSCs were incubated as described in the legend in Figure 3. IGFBP1 levels were measured at day 7 in the overnight conditioned medium of enMSCs using ELISA kit according to the manufacturer’s instructions. “−” indicates that IGFBP1 concentration is lower than ELISA detection limit (125 pg/mL), “+” that it is within detection linear range (125–16,000 pg/mL) and “++” that the IGFBP1 concentration is more than 16,000 pg/mL.

**Table 2:** Qualitative estimation of AREG amount in the conditioned medium of various human mesenchymal stromal cells and in cancer cell lines.

	AREG Secretion, pg/mL		
	− (<15)	+ (15–1000)	++ (>1000)
enMSC line 2804		√	
enMSC line 2602		√	
MSC-PL2			√
MSCWJ-1	√		
MSC-DP	√		
HeLa	√		
MCF-7			√

Cells were grown to confluence, and then the growth medium was changed and collected for ELISA analysis 2 days later. AREG levels were measured in the collected conditioned medium of indicated cell lines using ELISA kit according to the manufacturer’s instructions. “−” indicates that AREG concentration is lower than ELISA detection limit (15 pg/mL), “+” that it is within detection linear range (15–1000 pg/mL) and “++” that the AREG concentration is more than 1000 pg/mL.

## Data Availability

Not applicable.

## Abbreviations

The following abbreviations are used in this manuscript:

AREG	amphiregulin
BafA	bafilomycin A${}_{1}$
CHX	cycloheximide
EGF	epidermal growth factor
EGFR	EGF receptor
enMSC	endometrium-derived MSC
MSC	mesenchymal stromal/stem cells
TGF-α	transforming growth factor-α
